# Supplementary material for: The diagnostic accuracy of the GeneXpert ESBL-ampC prototype assay for rapid PCR-based detection of extended-spectrum beta-lactamase genes directly from urine
Source: Microbiol Spectr. 2023 Nov 14;11(6):e03116-23. doi: 10.1128/spectrum.03116-23 (PMC10715157; doi:10.1128/spectrum.03116-23)
Supplement: Supplementary appendix — Supplementary file I: Selection criteria for urine samples (planned and actual numbers). Supplementary file II: Protocol of ESBL and AmpC detection in Enterobacterales. Supplementary file III: Flow charts for processing discrepant ESBL and/or AmpC positive urine specimen and ESBL and AmpC negative urine specimen. Supplementary file IV: Whole genome sequencing methodology. [file spectrum.03116-23-s0001.docx]

**Supplementary files**

# Supplementary file I: Selection criteria for urine samples (planned and actual numbers)

| Selection criteria* | Planned numbers | Actual numbers |
| --- | --- | --- |
| ESBL culture positive Enterobacterales | 100 | 94 |
| MDRO – no ESBL | 20 | 26 |
| Chromosomal *ampC* producers (Group II Enterobacterales) | 22 | 25 |
| Bacterial mixed flora | 8 | 7 |
| Gram-positive mixed flora | 12 | 8 |
| Gram-negative mixed flora | 5 | 1 |
| Gram-negative cocci | 3 | 0 |
| Anaerobic mixed flora | 2 | 1 |
| No significant bacterial growth | 5 | 5 |
| Clarity of the urine sample | 10 | 11 |
| Epithelial cell grade ≥ 3 | 5 | 0 |
| *Escherichia coli* | 15 | 15 |
| *Klebsiella pneumoniae* | 10 | 9 |
| *Klebsiella* species, other than *K. pneumoniae* | 5 | 5 |
| *Proteus mirabilis* | 6 | 5 |
| *Proteus* species, other than *P. mirabilis* | 2 | 2 |
| *Citrobacter* species, other than *C. freundii* | 2 | 2 |
| *Raoultella* species | 2 | 2 |
| *Citrobacter freundii* | 3 | 3 |
| *Enterobacter cloacae* complex | 5 | 5 |
| *Enterobacter* species, other than *E. cloacae* complex | 2 | 0 |
| *Morganella morganii* | 5 | 5 |
| *Providencia rettgeri* | 2 | 2 |
| *Serratia marcescens* | 4 | 4 |
| *Acinetobacter* species | 1 | 1 |
| *Pseudomonas aeruginosa* | 5 | 4 |
| *Stenotrophomonas maltophilia* | 2 | 1 |
| *Staphylococcus aureus* | 4 | 4 |
| *Staphylococcus epidermidis* | 3 | 3 |
| Other *Staphylococcus* species | 1 | 1 |
| *Aerococcus* species | 4 | 4 |
| *Enterococcus faecalis* | 5 | 5 |
| *Enterococcus faecium* | 2 | 2 |
| *Corynebacterium* species | 1 | 1 |
| Haemolytic streptococci | 5 | 4 |
| *Actinotignum* species | 2 | 2 |
| Bifidobacterium | 1 | 1 |
| *Lactobacillus* species | 2 | 2 |
| *Gardnerella vaginalis* | 2 | 2 |
| *Candida albicans* | 2 | 2 |
| Other yeasts | 3 | 3 |
| Total | **300** | **279** |
| * It is possible that urine samples fit more than one selection criteria  ESBL = Extended-Spectrum Beta-lactamase; MDRO = multidrug resistant organism; Clarity of the urine sample = macroscopic observation of the degree of turbidity of the urine sample; Epithelial cell grade ≥ 3 = six or more squamous epithelial cells per field of view (enlarged 10 times). | | |

# Supplementary file II: Protocol of ESBL and *ampC* detection in Enterobacterales

**
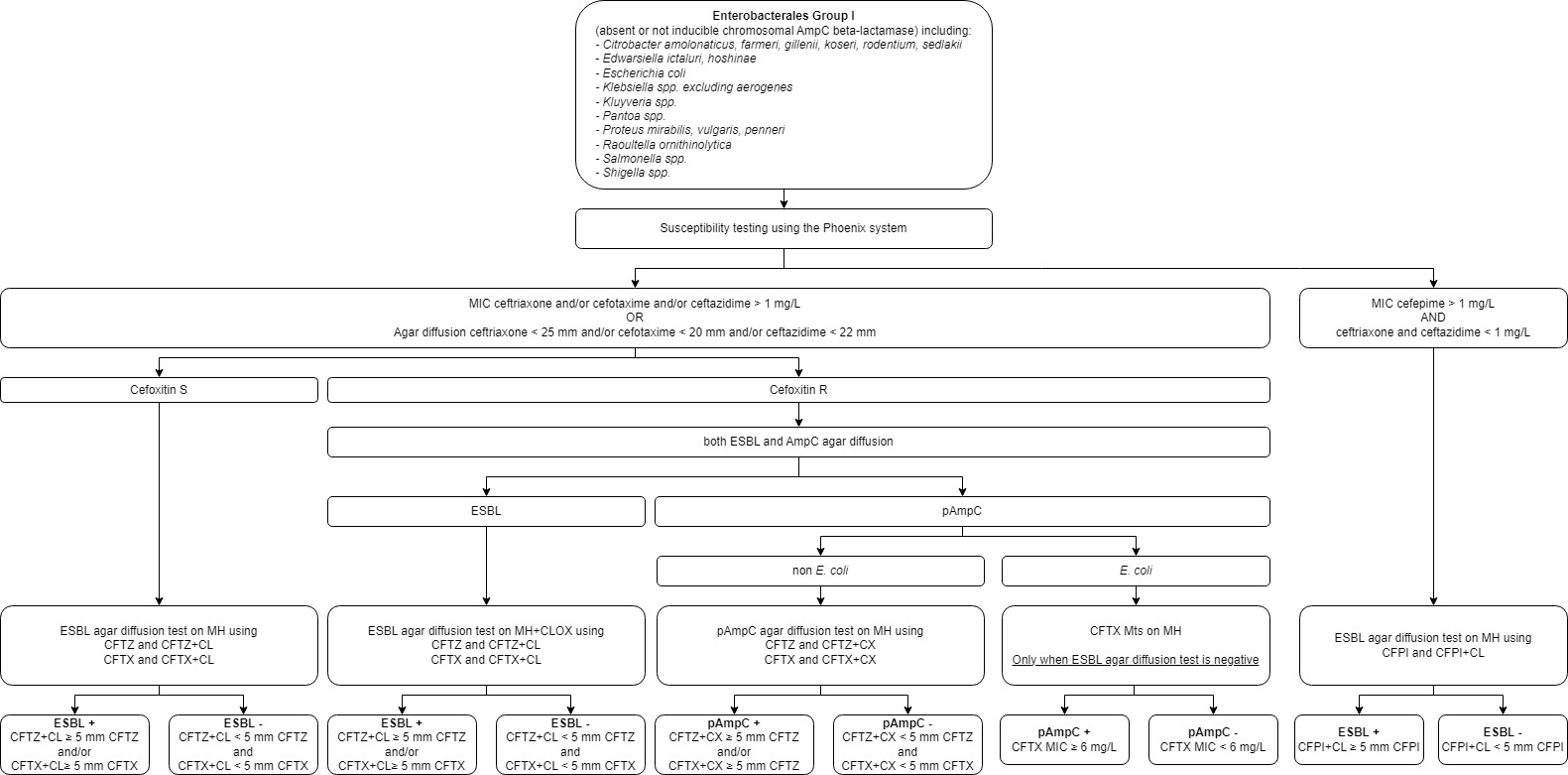
Hospital**


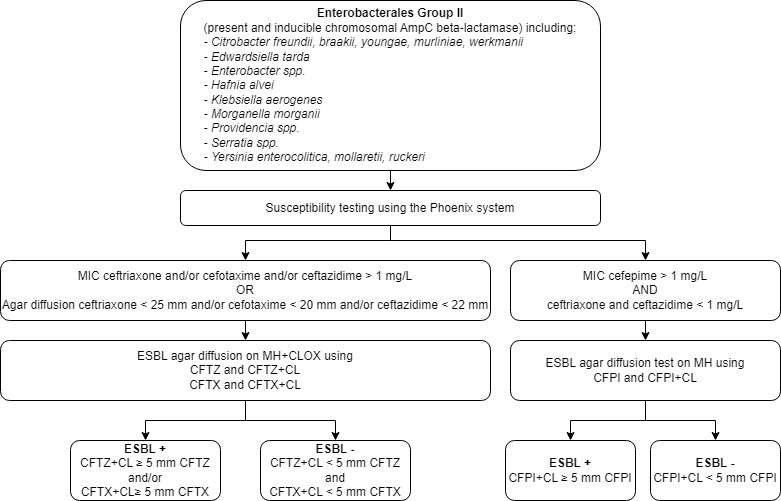
*Phenotypic ESBL confirmation*

CFTZ = ceftazidime; CFTZ+CL = ceftazidime + clavulanic acid; CFTZ+CX = ceftazidime + cloxacillin

CFTX = cefotaxime; CFTX+CL = cefotaxime + clavulanic acid; CFTX + CX = cefotaxime + cloxacillin

CFPI = cefepime; CFPI+CL = cefepime + clavulanic acid; MTS = MIC test strip

In case the MIC of cefotaxime, ceftriaxone and/or ceftazidime was >1 mg/L, phenotypic ESBL confirmation was performed using antibiotic discs containing ceftazidime (30 µg), ceftazidime/clavulanic acid (30/10 µg), cefotaxime (30 µg) and cefotaxime/clavulanic acid (30/10 µg) (BD, Diagnostic Systems, Sparks, MD, USA) on a Mueller-Hinton agar (Oxoid, Hampshire, United Kingdom). In case the MIC of cefotaxime, ceftriaxone and/or ceftazidime was ≤1 mg/L but the MIC of cefepime >1 mg/L, phenotypic ESBL confirmation was performed using antibiotic discs containing cefepime (30 µg) and cefepime/clavulanic acid (30/10 µg) (Bio-Rad, Marne La Coquotte, France). In case of possible ESBL and *ampC* production, a Mueller-Hinton agar containing cloxacillin was used (Oxoid, Hampshire, United Kingdom). Agars were incubated at 36 °C under O_2_ conditions for 16 - 20 h.

*Phenotypic ampC confirmation*

*ampC* confirmation was performed for group I Enterobacterales (not likely to express chromosomal-mediated *ampC*) if the cefoxitin MIC was >8 mg/L. For group II Enterobacterales, no *ampC* confirmation test was performed since inducible chromosomal-mediated *ampC* resistance genes are present in these species. For group I Enterobacterales non *E.coli* phenotypic *ampC* confirmation was performed using antibiotic discs containing ceftazidime (30 µg), ceftazidime/cloxacillin, cefotaxime (30 µg) and cefotaxime/cloxacillin (Rosco Diagnostica A/S, Taastrup, Denmark ) on a Mueller-Hinton agar (Oxoid, Hampshire, United Kingdom). For *E.coli*, phenotypic *ampC* confirmation was only performed in case of a negative ESBL confirmation test using a MIC test strip containing cefotaxime (MTS, Liofilchem, Italy) on a Mueller-Hinton agar (Oxoid, Hampshire, United Kingdom).


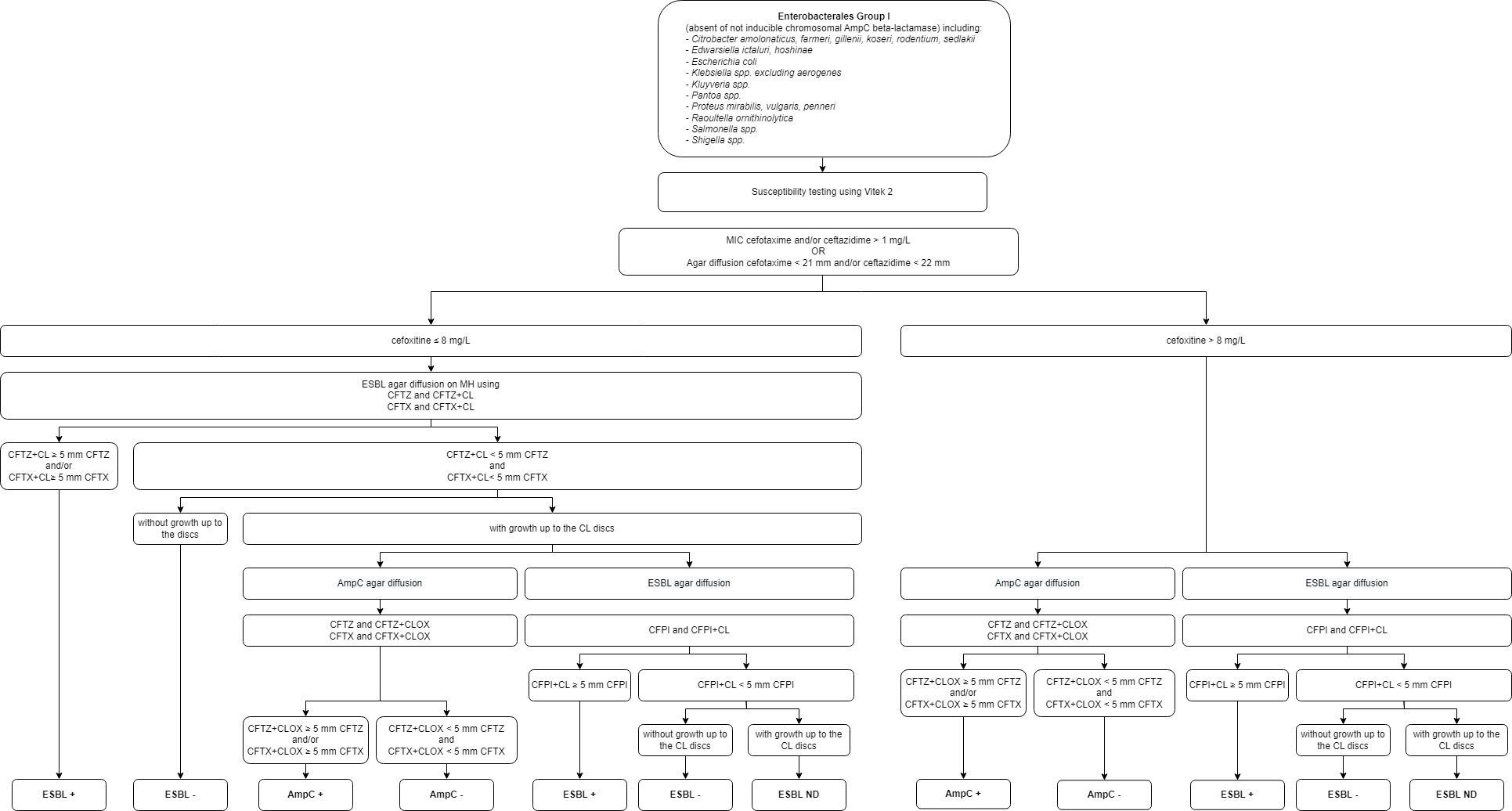
**Hospital 2:**


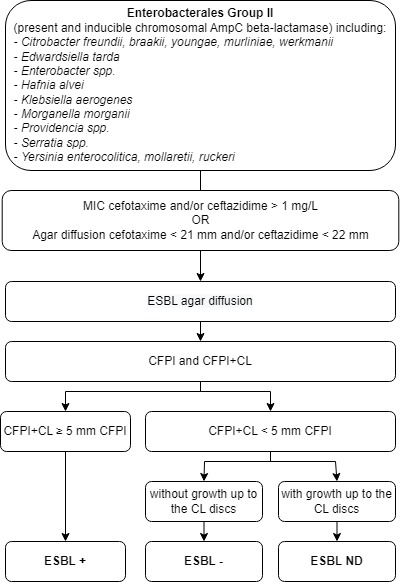


CFTZ = ceftazidime; CFTZ+CL = ceftazidime + clavulanic acid; CFTZ+CLOX = ceftazidime + cloxacillin

CFTX = cefotaxime; CFTX+CL = cefotaxime + clavulanic acid; CFTX + CLOX = cefotaxime + cloxacillin

CFPI = cefepime; CFPI+CL = cefepime + clavulanic acid

*Phenotypic ESBL confirmation*

Phenotypic ESBL confirmation was performed using antibiotic discs containing ceftazidime (30 µg), ceftazidime/clavulanic acid (30/10 µg), cefotaxime (30 µg) and cefotaxime/clavulanic acid (30/10 µg) (Liofilchem, Rosto degli Abruzzi (TE), Italy) on a Mueller-Hinton agar (Thermo Scientific, Vienna, Austria). In case of possible ESBL and *ampC* production (cefoxitin MIC >8 mg/L) antibiotic discs containing cefepime (30 µg) and cefepime/clavulanic acid (30/10 µg) (Liofilchem, Rosto degli Abruzzi (TE), Italy) were used. Agars were incubated at 36 °C under O_2_ conditions for 16 to 20 h.

*Phenotypic ampC confirmation*

If the cefoxitin MIC was >8 mg/L, phenotypic *ampC* confirmation was performed only for group I Enterobacterales using antibiotic discs containing ceftazidime (30 µg), ceftazidime/cloxacillin, cefotaxime (30 µg) and cefotaxime/cloxacillin (Liofilchem, Rosto degli Abruzzi (TE), Italy) on Mueller-Hinton agar (Thermo Scientific, Vienna, Austria).

Supplementary file III: Flow charts for processing discrepant ESBL and/or AmpC positive urine specimen and ESBL and AmpC negative urine specimen


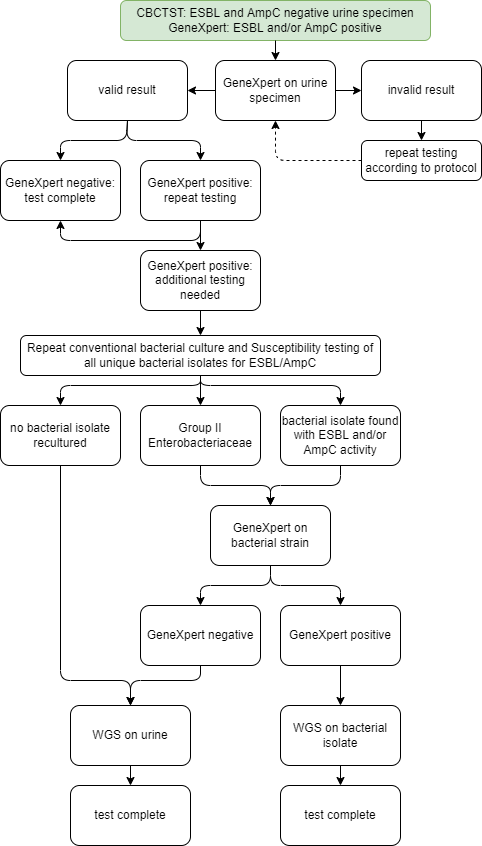


CBCTST: conventional bacterial culture techniques and susceptibility testing

WGS: whole genome sequencing


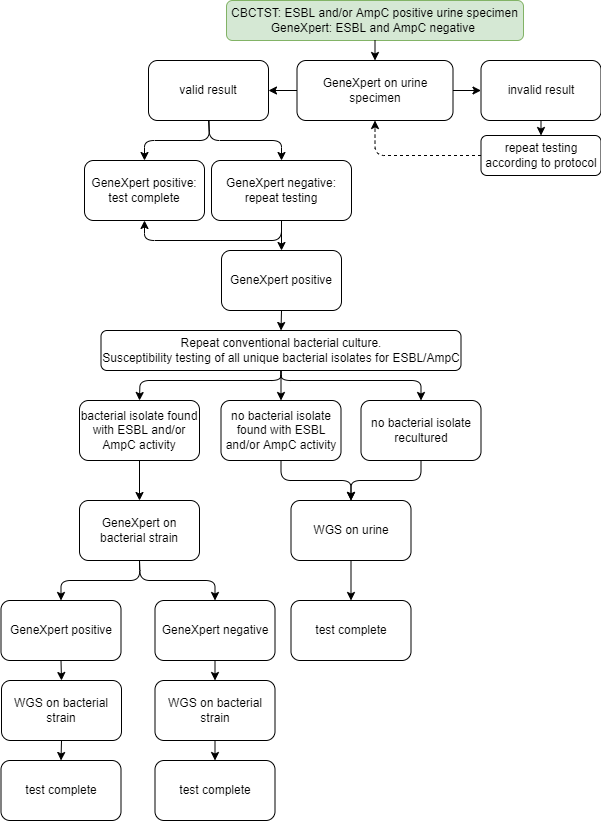


CBCTST: conventional bacterial culture techniques and susceptibility testing

WGS: whole genome sequencing

When analysing discrepancies between the microbiological culture results and the GeneXpert results, ocassionally urine samples were re-cultured (see the protocol above). In this context a different culture method was used to increase the chance that ESBL producing Enterobacterales that were present in low concentrations were detected as well. In short, a Columbia III agar plate, a MacConkey agar without salt plate and ESBL CHROM agar plate (bioMérieux SA, Marcy l’Etoile, France) were incubated at 36 °C under O_2_ conditions for 18 to 24 h. Thereafter, urine specimens were evaluated for the presence of bacteria. Identification and susceptibility testing were performed as described in the section *Routine microbiological urine culture* (main text) and confirmation of the ESBL and AmpC phenotype was performed as described in Supplementary File II. When bacterial isolates were tested on the GeneXpert, a 0.5 McFarland suspension was prepared. Subsequently, 10 µL was transferred into a 5 mL Sample Reagent Bottle and the assay was performed according to the manufacturer’s instructions.

# Supplementary file IV: Whole genome sequencing methodology

Whole Genome Sequencing (WGS) on bacterial isolates was performed as described by Shea et al. 2017 (1) for DNA isolation and as described by Coolen et al. 2019 (2) for library preparation and DNA sequencing. Raw reads were trimmed for quality and de novo assembled using CLC Genomics Workbench v. 21.0.5 (Qiagen Digital Insights, Aarhus, Denmark). Sequence analysis of bacterial isolates included identification of best matching reference using K-mer spectra, multilocus sequence typing (MLST), and drug resistance analysis using CLC Microbial Genomics Module v. 21.1 (Qiagen) and ResFinder database (downloaded on 2021-11-09, Center for Genomic Epidemiology). Assembly and taxonomic analysis of metagenomic samples was performed using the QC, Assemble and Bin Pangenomes workflow and the Taxonomic Profiling tool of CLC Microbial Genomics Module v. 21.1 (Qiagen).

References:

1. Shea J, Halse TA, Lapierre P, Shudt M, Kohlerschmidt D, Van Roey P, Limberger R, Taylor J, Escuyer V, Musser KA. 2017. Comprehensive Whole-Genome Sequencing and Reporting of Drug Resistance Profiles on Clinical Cases of Mycobacterium tuberculosis in New York State. J Clin Microbiol 55:1871-1882.
2. Coolen JPM, den Drijver EPM, Kluytmans JAJW, Verweij JJ, Lamberts BA, Soer JACJ, Verhulst C, Wertheim HFL, Kolwijck E. 2019. Development of an algorithm to discriminate between plasmid- and chromosomal-mediated AmpC β-lactamase production in Escherichia coli by elaborate phenotypic and genotypic characterization. Journal of Antimicrobial Chemotherapy 74:3481-3488.
